# A mixed-methods approach to understanding the perspectives, experiences, and attitudes of a culturally tailored cognitive behavioral therapy/motivational interviewing intervention for African American patients with type 2 diabetes: a randomized parallel design pilot study

**DOI:** 10.1186/s40814-022-01066-4

**Published:** 2022-05-21

**Authors:** Ronald M. Cornely, Vinita Subramanya, Ashley Owen, Robin E. McGee, Ambar Kulshreshtha

**Affiliations:** 1 Behavioral, Social, & Health Education Sciences Department, Rollins School of Public Health, Emory University, Atlanta, GA USA; 2 Department of Epidemiology, Rollins School of Public Health, Emory University, Atlanta, GA USA; 3 Department of Family and Preventive Medicine, Emory University School of Medicine, 4500 North Shallowford Rd.| Suite 134, Atlanta, GA 30338 USA

**Keywords:** African American, Type 2 diabetes, Cognitive behavioral therapy

## Abstract

**Background:**

African American (AA) adults are 60% more likely to be diagnosed with diabetes mellitus (DM) and experience more complications than non-Hispanic White adults. Cognitive behavioral therapy (CBT) has shown to be an effective modality for helping patients improve health behaviors and regulate emotional states. Motivational interviewing (MI) addresses participant engagement and motivation. Therefore, MI was combined with CBT as an approach to the process of learning using CBT skills to promote healthy lifestyle choices. We aimed to assess the effects of a culturally tailored CBT/MI intervention on glycemic control in AA participants and understand their perspectives, attitudes, and experiences while participating in this intervention.

**Methods:**

Using a randomized, parallel design pilot study (web-based group vs in-person group), 20 participants aged ≥ 18 years, identifying as AA and having a glycosylated hemoglobin (HbA1c) > 8%, were recruited. A CBT/MI intervention was administered in six sessions over 3 months. Participants completed baseline and follow-up assessments on measures for diabetes control (HbA1c), self-efficacy, generalized anxiety, depression, perceived stress, health-related quality of life, and cognitive ability. Post-CBT/MI intervention focus groups were conducted to determine patient perspectives regarding the intervention.

**Results:**

Fourteen participants completed the study, their mean HbA1c improved from 10.0 to 8.9% (*t*(26) = 0.5, *p*-value = 0.06). The Diabetes Distress Scale demonstrated decreased distress overall (*t*(26) = 2.6; *p*-value = 0.02). The Generalized Anxiety Disorder Scale demonstrated decreased generalized anxiety for all participants (*t*(26) = 2.2; *p* = 0.04). Themes identified in focus groups included (1) intervention group social support through information sharing, (2) mental health and personal identities in diabetes understanding and management, and (3) receptivity to CBT/MI intervention positively impacts self-efficacy through improved health literacy.

**Conclusion:**

This group-based, culturally tailored CBT/MI intervention for type 2 DM care was positively received by AA participants and helped improve diabetes control, as demonstrated by the change in HbA1c. There were additional benefits of social support through group interactions and a stronger sense of self-efficacy due to health education. A comprehensive treatment plan using a CBT/MI intervention may be useful in promoting healthy diabetes self-management.

**Trial registration:**

ClinicalTrials.gov, NCT03562767. Registered on 19 June 2018

## Key messages regarding feasibility

- What uncertainties existed regarding the feasibility?
  At the start of the trial, investigators were concerned about the feasibility of using a technology-based intervention in an older age group—in terms of both access and uptake. Another concern was the level of comfort among participants during discussions pertaining to their medical condition and barriers in achieving optimal health.
- What are the key feasibility findings?
  This study demonstrated that technology was not a barrier to the participation and retention of study participants. It indicated that group and web-based interventions were feasible and acceptable.
- What are the implications of the feasibility findings for the design of the main study?
  Future studies with a larger sample size, exploring the acceptability and effects of cognitive behavior therapy on diabetes mellitus control in African American people, are feasible.

## Background

In 2018, about 10% of the total US population was estimated to have diabetes mellitus (DM) [1]. According to the Centers for Disease Control and Prevention, diabetes has been documented to have a disproportionate impact on the African American (AA) population of the USA [2]. AA adults are more likely to receive a physician diagnosis of DM, have worse glycemic control, and have more DM-related complications compared to non-Hispanic White adults [3–6]. Risk factors for DM are also more prevalent in the AA population, such as a 1.3 times greater likelihood of obesity when compared to non-Hispanic White adults [7]. Other related risk factors seen in the AA population at higher rates include hypertension, high cholesterol levels, and smoking. Additionally, depression, anxiety, and diabetes-related specific concerns are common comorbidities in people with DM [8–10]. The relationship between DM and these co-morbidities is complex, since they are thought to influence each other and are affected by biological and psychosocial pathways [11]. The interaction between DM and related comorbidities adds a complex behavioral aspect of diabetes management, since it requires tasks with self-management components such as activity, diet, adherence and compliance with treatment regimens, self-monitoring of glucose levels, and making and keeping healthcare appointments. Further aggravating the impact of type 2 diabetes on AA adults are racial discrimination and low SES. These two factors are respectively associated with poorer health outcomes and a greater prevalence of diabetes [12, 13]. The greater risk and diabetes burden in AA patients suggests a need for tailoring interventions in this high-risk group.

Cognitive behavioral therapy is a form of psychotherapy consisting of a cognitive and a behavioral aspect that addresses thoughts, beliefs, and behaviors detrimental to self-care in chronic health conditions and replaces them with more helpful thoughts and behaviors [14, 15]. Psychological interventions such as cognitive behavioral therapy (CBT) have been shown as effective in overcoming behavioral barriers related to self-management and improved glycemic control among people with diabetes [16–18]. The CBT approach applies techniques to identify thoughts, beliefs, and behaviors that may be detrimental to self-care and replace them with more helpful thoughts and behaviors. Previous studies have found CBT to be useful in alleviating depressive symptoms associated with diabetes [19, 20]. Recent studies have also observed a moderate to large improvement in glycemic control with the implementation of CBT [19–22]. A sub-group analysis in a meta-analysis of studies using CBT among patients with diabetes found that the CBT had to be tailored to the population it was being implemented while being cognizant of the goals of treatment [22].

In this study, the CBT approach was modified by integrating motivational interviewing (MI) into the delivery of the intervention. A primary goal for integrating MI into the CBT approach was to support participants’ engagement in the intervention. In the MI theory [23], engagement is based on the effectiveness of the relationship between participants and facilitators. It is reflected in participants’ feeling understood, and it supports the participants’ motivation to learn and apply CBT content. Unfortunately, non-adherence to CBT skills practice is one of the most commonly cited reasons for CBT-based therapy failure [24]. With the goal of promoting adherence to the CBT skills practice regimen presented in this intervention, we integrated a focus on engagement and motivation by adding MI to the intervention. Therefore, a CBT/MI intervention was developed using MI to encourage patients’ acceptance and motivation to practice CBT skills that promote healthy lifestyle choices.

There are no studies that have examined the effects of a culturally tailored CBT/MI intervention in a group-based format among AA patients and examined its effects with uncontrolled diabetes. This pilot study aimed to assess the efficacy of a CBT/MI intervention in improving glycemic control among AA participants with Diabetes mellitus. It also aimed to understand participant experiences and evaluate their perspectives and attitudes toward this CBT/MI intervention.

## Methods

### Study design and setting

The Lifestyle Intervention Guidance for a Healthier Tomorrow (LIGHT) study was a randomized, parallel design pilot clinical trial. It was conducted at the Dunwoody Family Medicine Clinic, Emory University, Atlanta, GA, in 2019. The participants were administered a behavioral intervention (CBT/MI) program, from March to May 2019, with the administration of initial study scales shortly before beginning the study in March of 2019 and the collection of follow-up data in May 2019. It used a mixed methods approach with quantitative and qualitative study methods to assess glycemic control, perspectives, experiences, and attitudes of AA patients with diabetes mellitus. The trial was registered at ClinicalTrials.gov (identifier: NCT03562767).

### Study population (Fig. 1)

Potentially eligible patients were identified through chart reviews, response from community flyers, and online and phone advertising. Patients were contacted via letter or phone call from the investigator team with a description of the study. Eligible patients were recruited from the Emory Healthcare clinics and community in Atlanta.

**Fig. 1:**
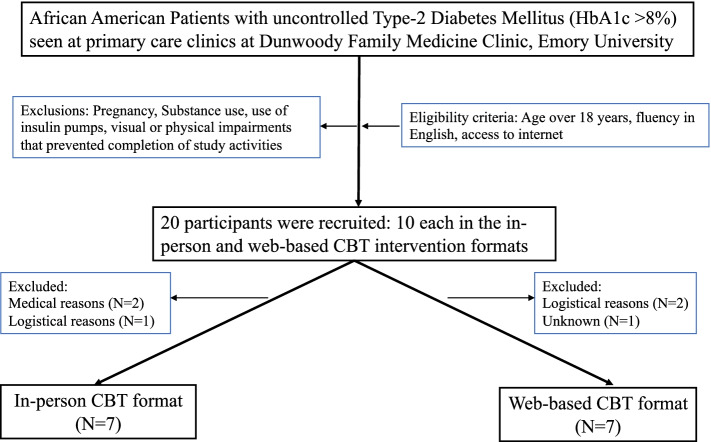
Participant flow diagram

At the first screening contact, participants gave verbal informed consent and were further interviewed by the study staff to determine eligibility. If patients are deemed eligible, they completed written informed consent and study questionnaires. Our study staff received clinical research coordinator training at Emory. The curriculum included training in culturally sensitive methods.

The eligibility criteria for this study included age ≥ 18 years, fluency in English, identifying as AA, and having a glycosylated hemoglobin (HbA1c) level of over 8% (an indicator of uncontrolled diabetes mellitus). Participants were excluded if they did not have internet access, used insulin pumps, were pregnant, actively misused substances (including alcohol), and had visual or physical impairments that did not allow them to complete study activities. Participants were followed up for a 3-month period. Of the initial 20 participants who were recruited, 14 participants completed the study with 7 in each intervention group. The six study participants who did not complete the study either failed to attend a sufficient number of intervention sessions or missed follow-up visits for post-study data collection. These study participants left after randomization. Two participants had diabetes-related complications and were hospitalized and thus unable to attend the study sessions. Two participants had difficulty with using Zoom despite training. One participant dropped out because of the timing of in-person sessions and weekend commitments. There is one participant who dropped out because of family challenges. All participants provided informed consent and research activities were approved by the Emory Institutional Review Board.

### Cognitive behavioral therapy motivational interviewing intervention

The intervention was developed and executed by a licensed psychologist with extensive experience in providing combined CBT and MI psychological treatment to African American patients. This individual is a member of the Motivational Interviewing Network of Trainers (MINT). As a MINT member, this psychologist is experienced in training clinicians to support participants’ engagement and motivation to make health behavior changes, including implementing CBT skills. The licensed psychologist trained the study facilitator using the Motivational Interviewing Treatment Integrity Coding Manual (MITI) 4.2.1 (Moyers T, Manuel J, Ernst D, Moyers T, Manuel J, Ernst D: Motivational interviewing treatment integrity coding manual 4.1, unpublished). Using the coding manual’s evaluation standards to determine competence, the study facilitator was determined to have met a “good” (highest category) threshold of clinician competence and proficiency in applying MI. In addition to ensuring that the intervention delivery training of the facilitator was MI congruent, the development of the intervention itself was crafted to apply MI in support of participant engagement.

Evidence suggests that, in general, adapted interventions outperform non-adapted interventions and are preferred by African American populations [25, 26]. Thus, we determined that developing behavioral health interventions for MCI-related cognitive and functional decline in ways that are culturally relevant to African American patients was important. To access expertise in cultural tailoring, we worked with an African American clinical psychologist and researcher who had expertise in both CBT and the cultural adaptation of evidence-based interventions for African American patients. The intervention team received implicit bias training, lectures, and ongoing consultation from our cultural tailoring content expert in order to learn about sociocultural considerations relevant to African American people and increase awareness of microaggressions that are commonly overlooked or dismissed [27]. The cultural tailoring content expert met virtually with the intervention team to provide feedback on drafts of each module on a bi-weekly basis. The goal of this feedback was to understand cultural considerations and strategies for integrating African American cultural values into the intervention.

Participants were randomized using computer-generated random numbers to one of two CBT/MI formats—a web-based and an in-person format. Both formats had six 1-h sessions, every 2 weeks for 3 months, focusing on food planning education (15 min) and culturally tailored CBT/MI presentations and interactive activities (45 min). The in-person format allowed for group interaction which was limited in the web format. The inclusion of both formats in a study assessing CBT/MI is a novel study design.

### Quantitative assessments

At baseline and during follow-up, participants were administered paper-based survey questionnaires that collected information on demographics. They also answered surveys on self-efficacy, generalized anxiety severity, depression severity, perceived stress, health-related quality of life (HRQoL), and cognitive ability. Sociodemographic information of interest included age, gender, education level, and marital status, all of which were self-reported. Participant body mass index (BMI) was used to determine obesity status (normal weight, overweight, obese). Additionally, glycosylated hemoglobin (HbA1c) information was obtained for all participants from electronic medical records and point of care testing, at baseline and during follow-up.

The measure of self-efficacy was collected using the Self-Management Resource Center’s Self Efficacy for Diabetes scale [28]. This 8-item scale assesses self-efficacy in diabetes self-management. A score closer to 10 is indicative of greater self-efficacy. The Diabetes Distress Scale is a 17-item, 6-point Likert, scale that evaluates the severity of emotional distress experienced by a person with diabetes [29]. It provides an overall score and one for each of the following dimensions: emotional burden, regimen distress, interpersonal distress, and physician distress. The patient’s responses associated with each of the four dimensions were summed then divided by the number of items in the given dimension. The mean of the responses to all 17 items was calculated to determine the overall distress experienced. A mean score of 3 (moderate distress) or higher serves as the indicator for clinical attention for study participants. Generalized anxiety in the 2 weeks prior to the scale’s administration was assessed using the Generalized Anxiety Disorder (GAD) Scale-7 [30]. It assesses the degrees to which participants feel anxious, nervous, or restless. A higher score indicates greater severity of anxiety. The Patient Health Questionnaire (PHQ)-9 scale assesses the severity of depression, a higher score is indicative of greater severity of depression [31]. The Perceived Stress Scale (PSS) was used to assess the extent to which participants identify situations in their life as stressful, within the past month [32]. A higher score suggests greater perceived stress. Health-related quality of life (HQRoL) was assessed using the RAND-36 survey [33]. It is a scale which assesses eight health concepts: physical functioning, role limitations caused by physical health problems, role limitations caused by emotional problems, social functioning, emotional well-being, energy/fatigue, pain, and general health perceptions [33]. Each of these health concepts are scored continuously (0–100), higher scores suggest a more positive perception of HRQoL. The Montreal Cognitive Assessment (MoCA) scale screens for cognitive dysfunction and assesses different cognitive domains through the performance of twelve different tasks [34]. A sum of item scores is used to determine cognitive ability, a score of ≥ 26 is considered “normal.”

### Qualitative assessments

Following the CBT/MI intervention, focus group sessions were conducted to evaluate the receptivity of the CBT/MI intervention among participants through semi-structured focus group discussion (FGD). FGDs provided the opportunity for participants to respond to each other’s feedback and find consensus about their experiences, especially regarding what worked well and what could be improved. Based on the intervention format to which they were randomized, participants were assigned to separate focus groups—one focus group for the web-based intervention and one group for the in-person intervention. These sessions were 25 min in length and were conducted in person in one of Emory University Healthcare System Dunwoody clinic’s conference rooms. The sessions were audio recorded by the interviewer, and there was a student collaborator present as a notetaker during each session.

The diffusion of innovations theoretical framework was used in designing the interview guide found in Appendix 1. This model is designed to communicate new knowledge to members of the target groups through four main elements: innovation, communication channels, social system, and time [35, 36]. A culturally tailored CBT/MI intervention was the innovation being assessed through web-based and in-person intervention formats. Communication channels were the culturally tailored materials presented to participants during the study. The social system is the target population: Black or African American patients with type 2 diabetes. The time is the incorporation of the study over a six-session intervention time frame. The diffusion of innovations model helped incorporate these key elements into the FGD guide to theoretically frame the information garnered from study participants.

The FGD questions aimed to capture participants’ perspectives, attitudes, and experiences with the CBT/MI intervention. The post-study focus group session evaluated the study acceptability and cultural congruence of the CBT/MI intervention through semi-structured group discussions. The focus groups aimed to assess compatibility, complexity, observability, and relative advantage of a culturally tailored CBT/MI for AA patients. It also aimed to assess the cultural sensitivity of the study.

### Data analysis

#### Quantitative analysis

Baseline characteristics of study participants were assessed using *t*-tests and chi-square tests, as needed. Baseline and follow-up assessments of quantitative scales were assessed for change in scores. This was done among the overall study population as well as by the intervention group. For continuous scales, the paired *t*-test were used to assess the change, and for categorical scales, the chi-square tests were used. Statistical significance was set at an alpha level of .05. Quantitative analyses were performed on the SAS 9.4 M software.

#### Qualitative analysis

Audio recordings were transcribed and imported into the MAXQDA analysis software. For each transcript, initial memos were developed to obtain familiarity of information in each interview. This was followed by the development of a codebook using inductive and deductive methods, which contains names and definitions of the codes and sub-codes that were developed. The deductive codes were developed based on the interview guide and purpose of the study (e.g., intervention receptivity and mindset). Inductive codes (e.g., balance/control) were included in the guide as they emerged from the collected data during analysis.

**Fig. 2:**
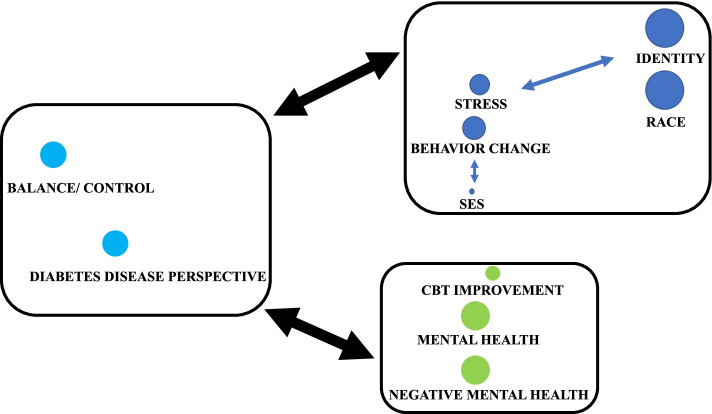
Disease perception, mental health, and identities (code map 3)

**Fig. 3:**
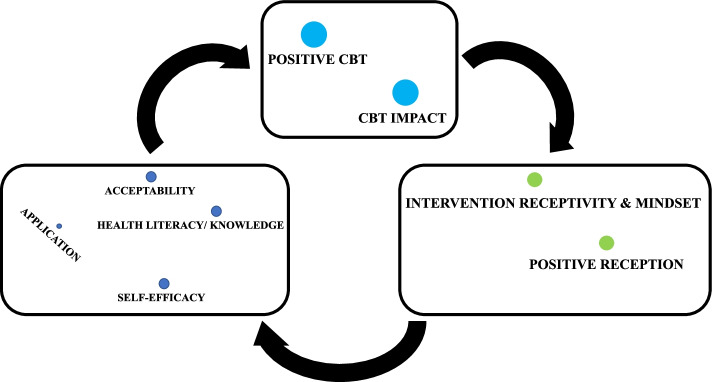
Receptivity, impact, and health literacy (code map 4)

The codebook can be accessed in Appendix 2. The transcripts were then coded by a student collaborator at the Rollins School of Public Health, whose coding techniques were discussed with the team and approved. Once coded, the codes and coded segments were reviewed to develop overarching themes describing related codes. Overarching themes were determined by assessing coding co-occurrence and proximity of codes through the MAXQDA code map function. A preliminary code map was used to assess the co-occurrence and coding similarities of all codes. The subsequent code maps (Figs. 2 and 3) were derived from the preliminary code map to determine the thematic threads of the study. Themes were further developed by exploring the differences and similarities in perspectives expressed within each intervention group. A second cycle of coding was completed by reviewing matrices of related codes and by comparing and contrasting themes of the two intervention groups. Quotes were identified that illustrated identified themes. For analytical purposes, participants were assigned IDs (1–14) to demonstrate the source of qualitative quotes to the reader by tagging each quote with the participants’ corresponding IDs. Assigning participant IDs assisted in demonstrating the breadth of perspectives incorporated into the qualitative analysis of the data. Participant ID assignments can be found in Appendix 3.

## Results

### Participant characteristics (Table 1)

Participants had a mean age of 56 ± 9.4 years, 79% identified as women (*n* = 11). About half of the participants had a college degree or higher and half reported being currently unmarried. The mean duration since a diagnosis of diabetes was 13 ± 5.3 years. BMI measurements categorized 64% of participants as obese. Demographic information can be found in Table 1.

**Table 1:** Participant characteristics at study baseline (*N* = 14)

Characteristic	Category	Frequency (***N***)	Percent (%)	Mean	Frequency (***N***), by group		Percent (%), by group		Mean		Standard error (in-person/web)	95% CI (in-person/online)	***P***-value (in-person/web)
		All Participants	All Participants	All Participants	In-Person	Web	In-Person	Web	In-Person	Web			
**Age (years)**	> 55	5	36	56.43	4	1	57	14	52	60.86	4.53	(− 0.98, 18.78)	0.07
	55+	9	64		3	6	43	86					
**Number of years with diabetes**	0 to > 5	2	14	13.30	0	2	0	29	14.86	11.5	2.82	(− 9.51, 2.80)	0.26
	6 to > 10	2	14		1	1	14	14					
	11 to > 15	5	36		3	2	43	29					
	16 to > 20	4	29		2	2	29	29					
	20 to > 25	1	7		1	0	14	0					
**Weight status**	Normal (BMI 18.5–24.9)	2	14	33.42	1	1	14	14	35.77	31.07	4.39	(− 14.26, 4.86)	0.31
	Overweight (BMI 25.0–29.9)	3	21		1	2	14	29					
	Obese (BMI ≥ 30.0)	9	64		5	4	71	57					
**Gender**	Man	3	21	(–)	2	1	29	14	(–)	(–)	(–)	(–)	(–)
	Woman	11	79		5	6	71	86					
**Marital status**	Single	7	50	(–)	4	3	57	43	(–)	(–)	(–)	(–)	(–)
	Married	4	29		1	3	14	43					
	Divorced	3	21		2	1	29	14					
**Highest level of education**	Less than a college degree	7	50	(–)	3	4	43	57	(–)	(–)	(–)	(–)	(–)
	College degree or more	7	50		4	3	57	43					

*Abbreviations*: *BMI* body mass index, *CI* confidence interval

### Quantitative results (Table 2)

The Self-Efficacy for Diabetes Scale results demonstrated a statistically insignificant decrease in scores for all participants, with a baseline mean score of 7.2 and a follow-up mean score of 7.1 (*t*(26) = 0.26; *p*-value = 0.80). The in-person and web-based groups also had statistically insignificant decreases in the mean group scores for self-efficacy, as demonstrated in Table 2. The Diabetes Distress Scale demonstrated decreased distress overall, from a mean at baseline of 2.5 to a follow-up mean of 2.0 (*t*(26) = 2.6; *p*-value = 0.02) and in each of the four dimensions it assesses: emotional burden, regimen distress, physician distress, and interpersonal distress. The physician distress and interpersonal distress did not have statistically significant differences between baseline and follow-up mean scores. The emotional burden dimension had a statistically significant decrease in the mean scores, with a baseline mean score of 2.9 and a follow-up mean score of 2.3 (*t*(26) = 2.6; *p*-value = 0.02). In-person participants also had a statistically significant decrease in emotional burden mean scores, as demonstrated in Table 2. The regimen distress dimension had a statistically significant decrease in the mean scores, with a baseline mean score of 3.1 and a follow-up mean score of 2.6 (*t*(26) = 2.6; *p*-value = 0.02). The web-based group also had a statistically significant decrease in regimen distress mean scores, as shown in Table 2. The Generalized Anxiety Disorder Scale had a statistically significant decrease in anxiety from mild anxiety to low anxiety for all participants, with a mean baseline score of 6.3 and a follow-up mean score of 4.2 (*t*(26) = 2.2; *p*-value = 0.04). The Generalized Anxiety Scale did not yield statistically significant differences in the in-person and web-based groups, as demonstrated in Table 2. Depression, as assessed by the PHQ-9 questionnaire, decreased by the end of the study but was not statistically significant for the study cohort and each intervention group (Table 2). Similarly, there was a statistically insignificant decrease in perceived stress levels in the study, as assessed by the Perceived Stress Scale (Table 2). More specifically, the in-person group had an increase in perceived stress (13.3 to 15.4) while the web format participants had a decrease in perceived stress (17.0 to 13.6). The only HRQoL domain of the RAND-36 survey that demonstrated a statistically significant increase was the pain health concept. A statistically significant increase for this health concept was demonstrated for participants overall as well as the in-person group. The overall mean score increased from 51 at baseline to 70 at follow-up (*t*(26) = − 3.8; *p*-value = 0.002), and the in-person group mean score increased from 39 at baseline to 64 at follow-up (*t*(26) = − 0.28; *p*-value = 0.01). Most of the remaining health concepts had increased mean scores from baseline to follow-up assessments, although these differences were not statistically significant (Table 2). In the paired *t*-test analyses, participant mean HbA1c improved from 10.0% at baseline to 8.9% during follow-up (*t*(26) = 0.5, *p*-value = 0.06) (Table 2).

**Table 2:** Quantitative scales score pre (baseline) and post (follow-up) cognitive behavioral therapy intervention

Scales	T2DM mean at baseline	T2DM mean at follow-up	***t***(26)	***P***-value	Max score
**SMRC self-efficacy**					10
All participants (*n* = 14)	7.21	7.10	0.26	0.80	
In-person (*n* = 7)	7.34	7.43	− 0.16	0.88	
Online (*n* = 7)	6.94	6.77	0.47	0.65	
**DDS total**					6
All participants (*n* = 14)	2.46	1.95	2.62	0.02*	
In-person (*n* = 7)	2.35	1.74	1.98	0.09	
Online (*n* = 7)	2.57	2.15	1.60	0.16	
**DDS emotional burden**					6
All participants (*n* = 14)	2.94	2.28	2.59	0.02*	
In-person (*n* = 7)	3.00	2.00	2.66	0.04*	
Online (*n* = 7)	2.89	2.56	1.00	0.35	
**DDS physician distress**					6
All participants (*n* = 14)	1.70	1.14	1.80	0.09	
In-person (*n* = 7)	1.39	1.07	1.12	0.31	
Online (*n* = 7)	2.00	1.21	1.42	0.21	
**DDS regimen distress**					6
All participants (*n* = 14)	3.11	2.55	2.6	0.02*	
In-person (*n* = 7)	2.71	2.11	1.47	0.19	
Online (*n* = 7)	3.51	2.99	2.80	0.03*	
**DDS interpersonal distress**					6
All participants (*n* = 14)	1.66	1.38	1.03	0.32	
In-person (*n* = 7)	2.00	1.33	1.92	0.10	
Online (*n* = 7)	1.33	1.43	− 0.25	0.81	
**GAD-7**					21
All participants (*n* = 14)	6.36	4.21	2.22	0.04*	
In-person (*n* = 7)	7.14	6.00	0.80	0.45	
Online (*n* = 7)	5.57	2.43	2.42	0.05	
**PHQ-9**					27
All participants (*n* = 14)	6.64	6.36	0.26	0.80	
In-person (*n* = 7)	7.29	7.71	− 0.35	0.74	
Online (*n* = 7)	6.00	5.00	0.52	0.62	
**PSS**					40
All participants (*n* = 14)	15.14	14.5	0.32	0.76	
In-person (*n* = 7)	13.29	15.43	− 1.37	0.22	
Online (*n* = 7)	17.00	13.57	0.95	0.38	
**RAND 36-physical functioning**					100
All participants (*n* = 14)	62.14	66.43	− 0.90	0.39	
In-person (*n* = 7)	53.57	60.00	− 1.19	0.28	
Online (*n* = 7)	70.71	72.86	− 0.26	0.80	
**RAND 36-physical problems limitations**					100
All participants (*n* = 14)	58.93	61.61	− 0.28	0.79	
In-person (*n* = 7)	42.86	57.14	− 0.88	0.41	
Online (*n* = 7)	75.00	66.07	0.92	0.39	
**RAND 36-emotional problem limitations**					100
All participants (*n* = 14)	69.05	61.91	0.67	0.51	
In-person (*n* = 7)	66.66	57.14	0.79	0.46	
Online (*n* = 7)	71.43	66.67	0.26	0.80	
**RAND 36-energy/fatigue**					100
All participants (*n* = 14)	47.14	52.86	− 0.99	0.34	
In-person (*n* = 7)	45.71	58.57	− 1.44	0.20	
Online (*n* = 7)	48.57	66.67	0.21	0.84	
**RAND 36-emotional wellness**					100
All participants (*n* = 14)	74.00	74.29	− 0.08	0.94	
In-person (*n* = 7)	77.71	74.86	0.55	0.60	
Online (*n* = 7)	70.29	73.71	− 0.66	0.53	
**RAND 36-social functioning**					100
All participants (*n* = 14)	61.61	75.00	− 2.03	0.06	
In-person (*n* = 7)	50.00	69.64	− 1.66	0.15	
Online (*n* = 7)	73.21	80.36	− 1.19	0.28	
**RAND 36-pain**					100
All participants (*n* = 14)	51.07	70.00	− 3.78	< 0.01*	
In-person (*n* = 7)	39.29	64.29	− 3.43	0.01*	
Online (*n* = 7)	62.86	75.71	− 1.95	0.10	
**RAND 36-general health**					100
All participants (*n* = 14)	55	55.71	− 0.13	0.90	
In-person (*n* = 7)	52.86	55.71	− 0.30	0.77	
Online (*n* = 7)	57.14	55.71	0.25	0.81	
**MOCA**					30
All participants (*n* = 14)	25.29	26.21	− 1.34	0.20	
In-person (*n* = 7)	25.14	25.71	− 0.55	0.60	
Online (*n* = 7)	25.43	26.71	− 1.33	0.23	
**HbA1c**					
All participants	10.0%	8.9%	0.5	0.06	

*Statistically significant difference indicated by a *p*-value less than 0.05

### Qualitative results

Themes identified from the qualitative data included the following: (1) intervention group social support through information sharing, (2) mental health and personal identities in diabetes understanding and management, and (3) receptivity to CBT/MI intervention positively impacts self-efficacy through improved health literacy.

#### Intervention group social support through information sharing

Participants in both intervention formats emphasized the importance of participating in the intervention as a means of social support which required intentional engagement with the intervention materials and between participants of the intervention. Two general ideas were expressed pervasively in the transcripts: (1) participants benefit most by sharing and receiving information and (2) participants were reassured by knowing that they were not alone in treating their diabetes. For this thematic thread, social support and information sharing were linked to interactions within the study groups but did not extend outside of the group. Reflections of the participants demonstrated perceptions of the importance of being open to the experiences of others and that day-to-day behaviors could be influenced by the information learned from group interactions. For example, one participant (participant ID 3) in the in-person group notably stated, “I learned a lot of information from each one of them cause each one of us had something different.” She later emphasized that, “You will learn something. You will learn something from somebody.” In seeking support from focus group members, the sentiment that participants were in a community where they could be understood was an important thematic thread. Participants were reassured by the communal experience of navigating diabetes management with others also inspired by the success they shared as a group. This sentiment was clearly expressed by one participant in the web-based group (participant ID 14) who stated, “I hear [somebody’s] victory so that encourages me.” The sense of belonging mirrored the effect of information sharing, as participants were willing to adjust behaviors to improve diabetes management. The support gained from a shared experience spurred participants to treat their diabetes in novel ways with the new information gained. Participants were in a space where they felt comfortable enough to honestly share instances of unhealthy behaviors, which illustrates the supportive environment that was promoted by the CBT/MI intervention. Instead of being criticized by fellow group members for admitting unhealthy behaviors, participants were instead encouraged to “do it in moderation.” Promoting moderation as a mechanism for more sustainable behavioral change was often mentioned in the transcripts and encouraged among participants to limit the intake of unhealthy foods. The community formed by study participants also provided study group members with accountability and positive reinforcement. One participant (participant ID 10) in the web-based group stated, “I know I was [going to] feel accountable when we had the meeting […] it definitely inspired me to do better,” clearly demonstrating the promotion of sustained behavioral change due to group accountability.

#### Mental health and personal identities in diabetes understanding and management

Study participants emphasized that the stresses associated with being AA or Black were unique. One in-person group participant (participant ID 7) stated, “African Americans may have some life stressors that other classes may not have.” Identifying the stressors associated with race was an important step in understanding underlying influences that contribute to effective behavioral changes. Race was not the only pertinent identity that participants discussed in relation to diabetes management. The same participant (participant ID 7) discussed how costs associated with proper diabetes management were linked to socioeconomic position when he stated, “The whole management [...] between medications and equipment [...]in terms of healthy eating [...] It can become taxing on the pocketbook.” The costs associated with proper diabetes management can impact beneficial behavioral changes when considering SES. Participant mental health also impacted their perception of diabetes. The mental health challenges of managing diabetes were shown to be linked to a lack of effective strategies for managing diabetes with reflections on how the CBT/MI intervention improved management techniques. Their reflections demonstrated that mental health was negatively impacted by having diabetes, which improved with the CBT/MI intervention. For example, the impact of the program was clearly expressed when a web-based group participant (participant ID 8) stated, “I would’ve went to eating […] comfort food […] and then I thought […] You know better. You just had the session on stress. […] That particular session did have an effect on what I ate during that stressful situation […].” Having a social network of people living with diabetes contributed positively to their outlook on diabetes management as this provided an outlet of negative emotions and an improved perspective of diabetes. Ultimately, a web-based participant’s (participant ID 10) reflection revealed that “the intervention […] made me realize that I wasn’t the only one,” emphasizing the importance of having a community to lean on. The code map that illustrates the visuospatial relation of the codes which informed this thematic thread can be found in Fig. 2.

#### Receptivity to CBT/MI intervention positively impacts self-efficacy through improved health literacy

Receptivity captured the positive dimensions of how study participants received study materials. The sentiment that positive reception was essential to a more successful participation experience was shared by many study participants. The positive reception of study materials with an improved outlook on living with diabetes resulted in participants retaining health information thus improving health literacy. Improved health literacy was most readily observed when a participant (participant ID 2) from the in-person cohort said, “You can go at it a different route, cause now you have more knowledge about what you’re dealing with”, identifying the benefit of health literacy to diabetes management. As a result, participant self-efficacy improved as they accepted and applied the knowledge shared with them. One web-based group participant (participant ID 9) shared that she acted on the information shared when she, “specifically went to the store and started buying vegetables instead of buying other things.” The intervention also increased awareness to life stressors to promote healthy coping strategies beneficial to diabetes management, thus providing another mechanism to maintain a positive outlook, informed decision-making. There was a cyclical trend of the positive reception of information which allowed participants to retain the learned information to be self-efficacious and apply it toward positive health outcomes. Per a web-based participant’s reflection (participant ID 14), participants were able to “recognize the problem […] apply those things they learned,” and were “prepared to deal with the situation” following the CBT/MI intervention. The code map that illustrates the visuospatial relation of the codes which informed this thematic thread can be found in Fig. 3.

## Discussion

This study evaluated the feasibility and acceptability of a CBT/MI intervention for African American patients with type 2 diabetes mellitus. It additionally explored the experiences of African Americans with type 2 diabetes and captured their perspectives concerning a culturally tailored CBT/MI intervention technique in managing their diabetes. An analysis was performed to determine how the intervention was received by participants and quantitative baseline and follow-up data was analyzed to determine if positive or negative perceptions were supported by health-related assessment tools. The study was conducted by independently assessing qualitative and quantitative data to gain an understanding of the implications that culturally tailored CBT/MI interventions have for Black or African American people in the USA. A mixed methods approach allowed the study team to explore how the information gleaned from the independent analysis of the quantitative and qualitative data could better inform study findings when considered concurrently. The results of the concurrent analysis illuminated two core concepts: (1) disease perception and perspectives on quality of life are interrelated, and (2) social support gained from CBT/MI interventions relates to perceived distress associated with diabetes management. This concurrent analysis revealed that a group-based, culturally tailored CBT/MI intervention for type 2 DM care was feasible, acceptable, and positively received by AA participants. There were benefits of social support through group interactions and a stronger sense of self-efficacy due to health education.

### Core concept: quality of life and disease perceptions

Participants reported increased mean scores, from baseline to follow-up assessments, in seven of the eight health concepts of the HRQoL scale. The concept of pain had a statistically significant increase during follow-up, for the overall group and the in-person intervention group. This is indicative of more positive perceptions of HRQoL in relation to pain associated with DM. The RAND-36 Emotional problems/limitations health concept is the only HRQoL health concept to differentially demonstrate decreased HRQoL overall and in both intervention groups. When coupled with the results of the depression scale, there exists a congruence in the observed trend in which participants have decreased health-related emotional capacity with mild levels of depression. These mixed results for HRQoL were particularly intriguing and warranted further consideration.

Concurrent analysis of the data emphasized the importance of considering how the unique stresses of being AA, expressed in the qualitative data, interacts with the quantitative mental health and HRQoL data. Participant awareness of their daily stresses, influenced by their racial identity, assisted in developing coping mechanisms that aid in diabetes management and may improve their perceived HRQoL. Awareness of these stresses may conversely lead to the cynicism that can contribute to other mental health concerns and perception of decreased HRQoL. The literature emphasizes that diabetes-related distress and depression can contribute to low glycemic control, treatment adherence behaviors, and poorer self-management [19–21]. However, research on social networks and diabetes management has shown that the quality of an individual’s social network could be an indicator of improved health outcomes in diabetes management [37]. The qualitative data indicated that providing a space with social support which disseminated pertinent health information could mitigate the mental health difficulties participants faced. Therefore, the community created in the respective intervention groups may have been beneficial to participant health outcomes during the study.

Sociological factors are also essential in holistically considering disparate health outcomes for AA adults with type 2 diabetes. Self-reported experiences of racial discrimination have been associated with poorer health outcomes in type 2 diabetes [12]. The effects of SES have also been shown to be associated with the prevalence of type 2 diabetes in the AA community [13]. The literature cites a strong inverse relationship between SES and diabetes incidence, citing that as income and educational level decrease, the diabetes risk increases two-fold [38]. Therefore, sociological factors independent of patient behavior may contribute to poor health outcomes. By gaining a better understanding of life stressors associated with their race and socioeconomic position, participants may have been able to develop better-coping strategies to manage their diabetes, further facilitated by their open-mindedness to behavioral change. The study’s qualitative data demonstrated the importance of SES in considering the costs associated with proper diabetes management. The associated additional costs result in higher levels of stress due to the added financial burden of diabetes care. The interaction of participant identities and stress when considering the behavioral change to improve diabetes outcomes was identified as a quintessential aspect of understanding the perspectives of AA patients.

A community was developed among participant groups where participants supported one another in positive diabetes management and mental health-related cognitions and behaviors. The intervention offered participants a skillset to cope with the stress of diabetes management and encouraged participants to have an active role in improving their quality of life. While there may have been an improved outlook on disease management, the stresses associated with daily diabetes management for an AA person are still likely to contribute to participants’ decreased emotional capacity to cope with diabetes in terms of mental health. Finding a community of support may help with the emotional burden of stresses associated with daily diabetes management. In fact, it is possible that the community created in the intervention groups was the factor of primary benefit to participant health outcomes during the study. Therefore, it could be helpful for future randomized control designs of this intervention to control for the impact of the group contact in order to better understand the impact of a culturally tailored CBT/MI intervention in the absence of group support.

### Core concept: emotional burden, distress, and social network support

The DDS demonstrated decreased distress overall and in each of the scale’s four dimensions: emotional burden, physician distress, regimen distress, and interpersonal distress. Emotional burden and regimen distress had scores greater than or equal to the clinically meaningful threshold of 3 at baseline and subsequent scores that decreased to levels below this threshold. These results suggest that the CBT/MI intervention appeared to lower distress associated with the diabetes management regimen, which could be more effective on a web-based platform given that this group was the only one with a statistically significant improvement.

Similarly, the intervention may be beneficial in alleviating distress associated with the emotional burden of diabetes, more so in an in-person setting given that they were the only group with a statistically significant improvement in this score.

It is important to note that a major documented comorbid factor to diabetes is depression. Studies have shown that depression and diabetes are likely to occur together twice as frequently as would be predicted simply by chance [9]. The incidence of depression has also been documented to be 24% higher in people with diabetes [39]. Not only are diabetes and depression more like to occur together, but they have also been shown to exacerbate symptoms. Epidemiological studies have demonstrated that the association between the two illnesses is bidirectional, which means that diabetes can impact the patient’s psychological well-being while depression can impact the severity of diabetes symptoms [40, 41]. More specifically, people with diabetes have longer lasting depressive episodes that occur more often than those in the general population [42]. The group-based design of the CBT/MI intervention was likely beneficial to patients who experienced the comorbid effects of diabetes and depression by providing a space to promote proper diabetes management and social support. The mental health, personal identities, and diabetes management themes revealed that participants had positive outlooks when in a social network of similar experiences. Group members demonstrated a level of comfort, such that they shared unhealthy eating behaviors. In turn, the group’s response was to approach diabetes treatment with a mindset of moderation, which was crucial in supporting participants.

Positive reinforcement and group accountability fostered candid conversations among group members and enabled participants to build a social support system. Health literacy disparities were addressed through the provision of health information from a trained health professional. Participant receptivity of study material was essential in addressing disparities in health literacy. An integrative review of the self-management of type 2 diabetes indicated that the perspectives and participation of patients is vital in the continued success of properly treating type 2 diabetes [43]. The participants’ willingness to learn and apply study information allowed the CBT/MI intervention to bolster their self-efficacy. Past research has also shown that combining medical nutrition therapy and self-management education improved patient outcomes with significant weight loss, HbA1c reduction, and a decrease in cholesterol levels [44]. Further research exploring the impact of diabetes self-management education demonstrated improved self-care, improved self-efficacy, and lower levels of psychological distress [45]. AA participants also demonstrated better health status with their improved psychological distress [45]. Therefore, promoting self-efficacy through this CBT/MI intervention was an appropriate approach to address the psychological distress associated with diabetes management.

Group member accountability and participant receptivity may have contributed to the decreased regimen distress observed in this setting of social support. Creating a communal component to diabetes management may be an effective method of improving the outlook of AA adults and mitigate the associated distress stemming from living with diabetes as a member of this racial group.

### Strengths and limitations

The study had a number of strengths. The provision of a culturally tailored CBT/MI intervention in two forms, an in-person format and a web-based format, has not been attempted previously, making it a very novel intervention approach. The study also addressed a significant gap in the existing literature by explicitly considering the perspectives of AA adults in chronic disease management. The study revealed unique aspects of health education and chronic disease management through the CBT/MI intervention, to aid participants in promoting positive health outcomes. The study provided participants with knowledge about psychological, behavioral, psychosocial, and social determinants of health for a more holistic treatment plan. Participants were empowered through information sharing and in finding social support among other study participants.

Our study was a pilot study, but it experienced loss to follow-up, potentially resulting in selection bias. This may affect the depth and richness of the data collected through the focus group discussions. Similarly, the length of the FGDs may have limited the richness of the data collected. Participants were given the time and space to express their perspectives, and probes were applied to provide ample opportunity to enrich the qualitative data collected. Despite the interviewer efforts, both group discussions were concluded in about 25 min, potentially limiting the depth of the qualitative data collected. It is important to note that combining qualitative data with quantitative data supplemented our understanding about experiences with the intervention. There was also a limited ability to explore qualitative differences between in-person and web-based groups. The FGD questions did not specifically inquire about the differences between the groups since it was not a focus of the study. A lack of consideration of the potential differences from administering the intervention on a web-based platform or in-person could have limited the observed impact of the study. Participants were provided the surveys to fill out on their own and assured anonymity. However, there is still a possibility that the self-reported data may have social desirability bias as they related to concepts of mental health, which is often stigmatized. Hemoglobin A1C was measured for a few participants using a point of care test which may have introduced measurement error. While the difference in the mean hemoglobin A1C scores was not significant, the data does indicate some promise for future studies. The information from focus groups may have come from participants who are more likely to actively engage with the study and may have resulted in an unintentional bias to be introduced toward more positive reflections.

## Conclusion

The study demonstrated that CBT/MI-based group intervention was feasible and acceptable in AA with uncontrolled type 2 diabetes. A CBT/MI-based group intervention led to positive changes in glycemic control (change in HbA1c from 10.0 to 8.9%), distress, and anxiety associated with diabetes mellitus. Our focus group analyses showed the benefits of social support through intervention group interactions and a stronger sense of participant self-efficacy due to health education and information sharing. Study results supported prior findings that mental/psychological health is an important factor in diabetes treatment and management, supporting the idea that diabetes is a multifactorial disease. Given the complex array of interacting components to diabetes, a more comprehensive treatment plan, like CBT/MI, may be useful in promoting healthy diabetes self-management. Future studies should consider expanding the study’s sampling frame to include a wider range of the AA population and similar tailored interventions targeting other marginalized groups. This approach would diversify the lived experiences of participants and provide more robust data to better understand how to support diabetes management in the AA community.

## Data Availability

Data can be made available on request, pending institutional and funding agency guidelines.

## Appendix

### Interview guide

Key questions: compatibility, complexity, observability, and relative advantage of a culturally tailored cognitive behavior intervention (CT-CB) for African American patients

1. What was your experience with the cognitive behavioral therapy intervention?
  - *Probe: What did you like about it? What didn’t you like about it?*
2. Have you ever participated in a similar intervention? Please tell us more.
  - *Probe: Did you prefer aspects of the other intervention more? Less? How about this intervention?*
3. What was confusing about the cognitive behavioral therapy intervention? What didn’t make sense to you?
  - *Probe: What about it was confusing? How could we have better communicated with you?*
4. How has the cognitive behavioral therapy intervention impacted how you manage your diabetes, if at all?
  - *Probe: Did you feel like you had more control of your diabetes? Did you feel like you had less control of your diabetes?*
5. How did the material presented from the therapy align with your daily activities?
  - *Probes: Is it easy to incorporate? What is difficult to incorporate? How would it have been easier to make it as part of your daily life? Have you done something like this before?*

Key questions: cultural sensitivity of the study

1. How could the study improve in making the intervention and experience more comfortable for African American patients?
  - *Probe: Did you ever feel uncomfortable? Why?*
2. How did the study staff make you feel during the intervention?
  - *Probe: Words of encouragement? Attitudes? Demographics?*
3. How did the staff account for your beliefs and identity when presenting the intervention material?
  - *Probe: Words? Actions? Intervention specific components?*

Closing questions/conclusion

1. If you could advise other African American patients on how to navigate these experiences what would you say?
2. Do you have any final thoughts you would like to share?

## Appendix

**Table 3:** Code system

Code #	Code	Sub-code	Definition	Example quotes
1	Diabetes disease perspective		How participants view diabetes as a disease and how they describe it when discussing the intervention. Discussions about how diabetes relates to them are also useful.	“This whole experience is just a rollercoaster. Every day you do everything right, you take your blood sugar levels and it’s all over the map”
1.1		Balance/control	When participants discuss dealing with aspects of their lives in relation to diabetes. Reference could be to diabetes as a disease, navigating the learning curve of diabetes, or managing diabetes.	“Sometimes I’m good, sometimes I’m not so good. Sometimes the experiences are good, sometimes they’re not good.”
1.2		Behavior change	Mention of action taken to improve self-management of diabetes which includes a behavioral change by participants.	“In my life I found out that I have a lot more power to choose what I do”
2	Identity		Any mention of how participants view themselves or how society may view them.	“Many of us could relate to it, you know, we’ve experience it or we’re living it”
2.1		SES	Experiences that participants describe which are related to the individual’s socioeconomic status (positive or negative). This may include costs of treatment and living expenses.	“It can become, it can become taxing on the pocketbook”
2.2		Race	Any mention of race as a social construct, whether related to the personal identity of the participant or in relation to society in general.	“So, this disease impacts African Americans somewhat differently based on culture”
3	Health literacy/knowledge		Mention of information learned from the CBT intervention.	“You know, the materials made you think about things that you hadn’t thought about”
4	Coping		Mentions of how the participant has been able to handle the stresses of diabetes management.	“that particular session did have an effect on what I ate during that stressful situation”
5	Information sharing		Sharing of information with others concerning diabetes management and coping mechanisms.	“I learned a lot of information from each and every one of them”
5.1		In-group	Sharing of information in a given treatment group.	“And we learned some things from each other [Group members verbally agreeing]”
5.2		Public	Discussions of disseminating information to the public.	“They gotta get it out. In, you know, be it in spurts or increments”
6	Self-efficacy		Reflections on how participants have acted to improve their health when managing their diabetes as a result of the information learned from the CBT/MI intervention.	“So, I specifically went to the store and started buying vegetables instead of buying other things”
7	Acceptability		Mentions of how well received the information was by the group and how they believe it would be received by other African American/ Black people.	“I think the materials were really helpful.”
7.1		Application	Mention of acceptability of material that leads to the application of learned material.	“Umm, problem solving, you know, um, and just kind of trying to give you options and things to think about, uh, day-to-day”
8	Agreement		When participants express that they have had the same, or similar, experiences. Verbal and non-verbal cues in the transcript should be coded.	“I think because I feel like it’s more people that are around that are going through some of the same things.”
8.1		Treatment agreement	When participants express similar experiences in relation to treatment (good or bad)	“They’ve been eye opening. They’ve been, they’ve been very direct [Other participant: Mhm hmm] and it’s kind of made me think out of the box”
9	Social support network		Any mention of being encouraged or finding refuge from others.	“We really had a great group”
9.1		In-group support	Encouragement or refuge from the intervention group.	“Well the intervention made me feel like, well I’m not the only one”
9.2		At-home/societal support	Encouragement or refuge from people outside of the intervention group.	No data coded.
10	Mental health		Any indication of psychological status from study participants.	“I feel a reduction in my life, and does it ever get any better?”
10.1		Positive mental health	Any indication of positive experiences related to the patient’s state of mind and diabetes management.	“help you evolve, you know, into a better person. So, it’s been, it’s been real good.”
10.2		Negative mental health	Any mention of desperation or loss of hope from participants.	“I would say depressed sometimes.”
11	Stress		Any mention of psychological or emotional distress from participants.	“And something that really just “stress me out , and automatically I would’ve went to eating something that, you know, people say comfort food or whatever.”
12	Intervention receptivity and mindset		Discussions about how well-received study materials and intervention were by participants and what their state of mind was.	“It’s making me feel like I can grow, you know, dealing with them and they’ve changed a lot of things, you know, as far as medication and all.”
12.1		Positive reception	Discussions of when intervention materials were well received.	“One thing it helped me with is periodically I know I was gonna feel accountable when we had the meeting, so it kind of inspired me to do better”
12.2		Negative reception	Discussions of when intervention materials were not well received.	No data coded.
13	Environmental and ecological factors		Discussions about how participant environment affected the use of materials from the study intervention by participants.	“Go to a cooking class and learn how to make the foods that you like in a different way”
14	CBT/MI impact		Any mention of how CBT/MI has contributed to the treatment of disease or how the information has been received by participants.	
14.1		Positive CBT/MI	How CBT/MI has contributed to participants positively.	“It was helpful.”
14.2		Negative CBT/MI	How CBT/MI has contributed to participants negatively.	No data coded.
14.3		CBT/MI improvement	How CBT/MI can be improved.	“I think that we could’ve gotten suggestions. Recipes or things to avoid.”

Table 3

## Appendix

**Table 4:** Participant ID assignments

Intervention	Participant	Participant ID
In-person	Woman 1	1
	Woman 2	2
	Woman 3	3
	Woman 4	4
	Woman 5	5
	Man 1	6
	Man 2	7
Web-based	Woman 1	8
	Woman 2	9
	Woman 3	10
	Woman 4	11
	Woman 5	12
	Woman 6	13
	Man 1	14

Table 4

## Abbreviations

AA: African American
DM: Diabetes mellitus
CBT: Cognitive behavioral therapy
MI: Motivational interviewing
HbA1c: Glycosylated hemoglobin
SES: Socioeconomic status
BMI: Body mass index
GAD: Generalized anxiety disorder
PHQ-9: Patient Health Questionnaire (PHQ)-9
PSS: Perceived Stress Scale
HRQoL: Health-related quality of life
FGD: Focus group discussion
